# Health impacts of Obesity

**DOI:** 10.12669/pjms.311.7033

**Published:** 2015

**Authors:** Shirin Djalalinia, Mostafa Qorbani, Niloofar Peykari, Roya Kelishadi

**Affiliations:** 1 Shirin Djalalinia, MSc PhD Candidate, Non-communicable Diseases Research Center, Endocrinology and Metabolism Population Sciences Institute, Tehran University of Medical Sciences, Tehran, Iran, and Endocrinology and Metabolism Research Center, Endocrinology and Metabolism Research Institute, Tehran University of Medical Sciences, Tehran, Iran, and Development of Research & Technology Center, Deputy of Research and Technology, Ministry of Health and Medical Education, Tehran, Iran.; 2 Dr. Mostafa Qorbani, PhD, School of Medicine, Community Medicine Department Alborz University of Medical Sciences, Karaj, Iran, and Non-communicable Diseases Research Center, Endocrinology and Metabolism Population Sciences Institute, Tehran University of Medical Sciences, Tehran, Iran.; 3 Niloofar Peykari, MSc PhD Candidate, Non-communicable Diseases Research Center, Endocrinology and Metabolism Population Sciences Institute, Tehran University of Medical Sciences, Tehran, Iran, and Endocrinology and Metabolism Research Center, Endocrinology and Metabolism Research Institute, Tehran University of Medical Sciences, Tehran, Iran, and Development of Research & Technology Center, Deputy of Research and Technology, Ministry of Health and Medical Education, Tehran, Iran.; 4 Prof. Roya Kelishadi, MD, Child Department of Pediatrics, Child Growth and Development Research Center, Research Institute for Primordial Prevention of Non-communicable Disease, Isfahan University of Medical Sciences, Isfahan, Iran.

**Keywords:** Overweight, Obesity, Health Impact

## Abstract

The aim of this communication is to provide some evidence linking the overweight/obesity and their impacts on different dimensions of health. We reviewed the related studies published from 1990 up till now through PubMed Central/Medline, which provide evidence linking obesity with health related issues. It is a risk factor for metabolic disorders and leads to serious health consequences for individuals and burden for the health care system as a whole. Literature search showed that it is related to at least 18 co-morbidities which are attributable to overweight and obesity. Moreover obese individuals more often suffer from significant joint pains, disorders and it also has social as well as psychological impairments. It is high time that countries facing the problems of obesity initiate some intervention measures to monitor and control this growing epidemic.

## INTRODUCTION

The dramatic increase in the prevalence of overweight and obesity in most countries has been of great concern globally.^1^^-^^3^ This is estimated to be the cause of more than 3.4 million deaths, 4% of Years of Life Lost (YLL), and at least 4% of Disability-Adjusted Life Years (DALYs) all around the word.^2^ However, despite the urgency of this problem, there are still some noticeable gaps in what is known about this subject. For instance prevalence of obesity is most often estimated based on surveys or population studies. Not only that data on prevalence and trends are based on measurements of weight rather than the body fat.^4^

This increase in body mass presents public health challenges because of attractive physical appearance of thin bodies, and poor health outcomes of overweight and obesity.^1^^,^^3^ Health condition of obese persons' is most often worse than people with normal weight and the life span of obese people is on average is shorter by two years.^5^

## PHYSICAL HEALTH IMPACTS

Some of the co-morbidities related to overweight and obesity include cancers (cancers of breast, endometrial, ovarian, colorectal, esophageal, kidney, pancreatic, prostate), Type 2 diabetes, hypertension, stroke, Coronary Artery Disease, Congestive Heart Failure, asthma, chronic back pain, osteoarthritis, pulmonary embolism, gallbladder disease, and also an increased risk of disability. All this leads to more than three million deaths worldwide annually.^3^^,^^6^

There is also consistent association between overweight and obesity in childhood and adolescence with increased risk of both premature morbidity and mortality particularly cardio-metabolic morbidity.^7^

It is estimated that in industrialized countries, disability due to obesity-related cardiovascular diseases will increase, under an increasing trend.^2^^,^^8^ The main reason being increased survival of these patients with cardiovascular diseases in these countries. Moreover because of insufficient insulin supply in these countries, disability due to obesity-related and type 2 diabetes will also increase due to arteriosclerosis, nephropathy and retinopathy.^8^ Yet another related health problem due to increasing prevalence of obesity will be the number of years that patients suffer from obesity-related morbidity and disability which would also increase significantly.^8^

Studies have confirmed that obesity is a major public health problem which results in decreased life expectancy especially in younger age groups.^1^^,^^2^ BMI itself, even without considering the other anthropometric measures (e.g., waist circumference, waist-to-hip ratio), is a strong predictor for overall mortality. This estimation includes both values, above and below the expected level of about 22.5-25 kg/m^2^. Above this defined range the progressive increase in mortality is mainly related to cardiovascular disease. At the range of 30-35 kg/m^2^, mostly, median survival is reduced by 2-4 years; whereas at 40–45 kg/m^2^, it is reduced by 8-10 years. The expected increase in mortality below 22·5 kg/m^2^ is not clearly explained.^9^

 Studies also confirm that overweight and obesity is a major problem for minority population than for whites, in poor as compared to the rich and in women as compared to men.^10^

Overweight and obesity also carry a considerable health burden and will have a significant impact on health expenditures.^6^ Obesity has a strong association with the occurrence of chronic medical problems, impairment of health-related quality of life, and increasing the health care and medication spending,^6^^,^^10^^,^^11^ the related health care costs for obesity-related problems, for both individuals and health care systems, are substantial.^12^

## IMPACT ON MENTAL HEALTH

Relationship between obesity and mental health disorders is not clear.^13^ However, overweight is a stigma and the obesity discrimination can lead to some mental disorders. Scientific evidence lays emphasize on an increasing risk of low self-esteem, mood disorder, motivational disorders, eating problems, impaired body image, interpersonal communication problems and all these directly or indirectly affect the quality of life.^10^^,^^14^

On the other hand in some cases, experiencing the obesity discrimination has lead to the development of psychopathology and poor health behavior that through a vicious cycle, will enhance their overeating, bulimia, or other related problems.^14^

Some studies have revealed that obesity in both men and women increase the risk of poorer sexual health.^15^ Obese individuals, attribute this to their appearance and their weight, and encounter frequent difficulties in their sexual activities.^15^^,^^16^ Sexual activity and sexual health outcomes such as sexual satisfaction, unintended pregnancy, and abortion have been mentioned as relevant issues.^15^^,^^16^ Sexual quality of life is particularly impaired for obese women who are also faced with complexity of the therapeutic procedures. ^15^

As such we need to emphasize on more comprehensive population based studies to find out the impact of overweight and obesity on different aspects of mental health including mood disorders, communication problems, self satisfaction and its effects on sexual health besides different aspects of quality of life.^15^^,^^16^

## IMPACT ON SOCIAL ASPECTS

Consequences of obesity-related physical co-morbidity includes psychological impairments and stigmatization experienced by obese patients.^14^^,^^17^

The overweight stigma and attributable discrimination is documented in all the key areas of living, including growth and development, educational process, employment structure, and provision of health care.^18^ The obese individuals are most often ridiculed by their teachers, physicians, and public. At times they also suffer from discrimination, ridicule, social bias, rejection, and humiliation.^14^^,^^18^ Even specific obesity diagnostic or therapeutic procedure such as related anthropometric assessments could potentially affect their care givers professional attitude and subsequent clinical evaluation and service provision for obese persons when they are seeking care.^18^

Weight-related discrimination, by itself is related to poor health behavior such as pathological overeating, binge eating or even sedentary life and decreased physical activity that in turn leads to greater weight gain. This vicious cycle, again strengthens the risk of exposure to weight-related discrimination.^18^

## SPIRITUAL ASPECTS

Studies on obesity and its consequences on spiritual health are very limited. Exploratory evaluation on the relationship between emotional eating and spiritual well-being showed that lower levels of spiritual well-being is correlated with higher levels of emotional eating specially in women. There is some evidence that, emotional eating contributes to impaired nutritional behaviors such as higher caloric intake, binge eating, and bulimic eating desires. Some other studies have emphasized on the important role of education which leads to better spiritual perception.^19^^,^^20^

## POLICY CONSIDERATIONS

Considering the importance of health risks of overweight and obesity and its increasing prevalence all over the world there is a need for well defined programs on control and prevention which should be a priority on the political health agenda.^8^ If this increase in its prevalence continues, it could lead to serious health related outcomes and consequences. However, so far only a few comprehensive preventive programs have been developed with little reported success. 

**Fig.1:**
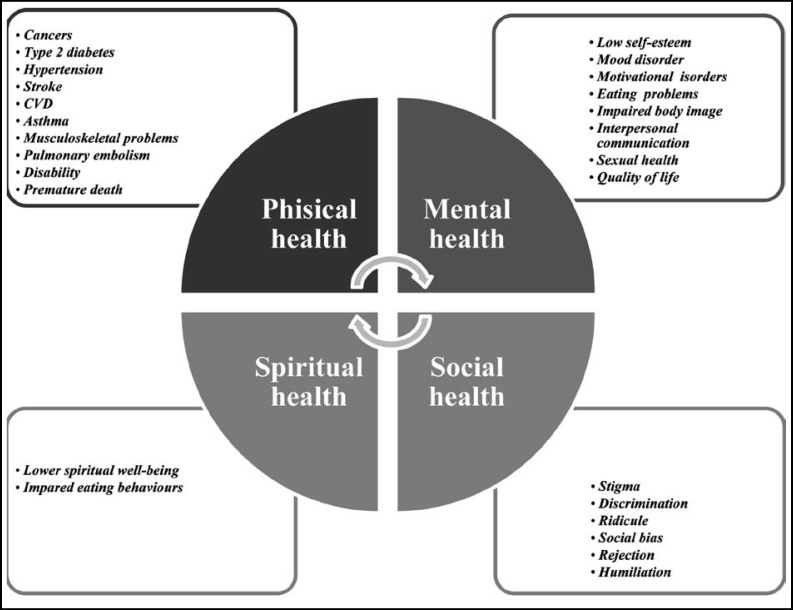
The most common consequences of obesity on the main domain of health.

The contributions of promoting physical activity, changes in food types and calorie consumption, detecting and controlling the eating behavioral impairments, and other related factors of overweight and obesity prevalence are some of the issues which need further research.^2^^,^^14^^,^^20^

## CONCLUSION

Overweight, obesity and their impacts in different dimensions of health must be considered as one of the most important public health priority. There is a need for comprehensive strategies for prevention and control of this epidemic.
